# Lactic Acid Induces Aberrant Amyloid Precursor Protein Processing by Promoting Its Interaction with Endoplasmic Reticulum Chaperone Proteins

**DOI:** 10.1371/journal.pone.0013820

**Published:** 2010-11-03

**Authors:** Yiwen Xiang, Guilian Xu, Kirsten A. K. Weigel-Van Aken

**Affiliations:** 1 Division of Cellular and Molecular Therapy, Department of Pediatrics, University of Florida College of Medicine, Gainesville, Florida, United States of America; 2 Powell Gene Therapy Center, University of Florida College of Medicine, Gainesville, Florida, United States of America; 3 Department of Molecular Genetics and Microbiology, University of Florida College of Medicine, Gainesville, Florida, United States of America; 4 Department of Neuroscience, University of Florida College of Medicine, Gainesville, Florida, United States of America; Massachusetts General Hospital and Harvard Medical School, United States of America

## Abstract

**Background:**

Lactic acid, a natural by-product of glycolysis, is produced at excess levels in response to impaired mitochondrial function, high-energy demand, and low oxygen availability. The enzyme involved in the production of β-amyloid peptide (Aβ) of Alzheimer's disease, BACE1, functions optimally at lower pH, which led us to investigate a potential role of lactic acid in the processing of amyloid precursor protein (APP).

**Methodology/Principal Findings:**

Lactic acid increased levels of Aβ40 and 42, as measured by ELISA, in culture medium of human neuroblastoma cells (SH-SY5Y), whereas it decreased APP metabolites, such as sAPPα. In cell lysates, APP levels were increased and APP was found to interact with ER-chaperones in a perinuclear region, as determined by co-immunoprecipitation and fluorescence microscopy studies. Lactic acid had only a very modest effect on cellular pH, did increase the levels of ER chaperones Grp78 and Grp94 and led to APP aggregate formation reminiscent of aggresomes.

**Conclusions/Significance:**

These findings suggest that sustained elevations in lactic acid levels could be a risk factor in amyloidogenesis related to Alzheimer's disease through enhanced APP interaction with ER chaperone proteins and aberrant APP processing leading to increased generation of amyloid peptides and APP aggregates.

## Introduction

Early-onset, autosomal dominant, familial Alzheimer's disease (AD) is caused by mutations in proteins that participate in the genesis of amyloid peptides. Mutations in APP, the progenitor of Aβ peptides, are a documented cause of AD as are mutations in 2 proteins, presenilin 1 and presenilin 2, which are components of a proteolytic enzymatic complex that is directly involved in the processing of APP[1]. Multiple studies have established that the net effect of these mutations is to increase the relative proportion of Aβ42 peptide that is generated by APP processing or to increase overall Aβ42 production [2]. Thus, these familial forms of the disease establish increased Aβ42 production as one mechanism by which the onset of AD can be hastened. However, few cases of AD are either early-onset or inherited. For the vast majority of these cases, the causative factors, other than aging, are less clear.

A common characteristic of many metabolic and vascular diseases is increased production of lactic acid. High lactate levels are found in affected tissues of individuals with disease caused by mitochondrial mutations, leading to mitochondria dysfunction [3]. Stroke and cerebral ischemia are associated with stimulation of glycolysis due to low availability of oxygen, resulting in increased levels of lactate in the brain [4]. Relevant to the present study, the levels of lactate in the cerebrospinal fluid (CSF) of AD patients has been reported to be elevated [5] and one of the enzymes critical to the production of Aβ peptides, β-amyloid cleaving enzyme (BACE1), is strongly influenced by pH, with an optimal pH well below 6.0 [6]. Thus lactic acid has the potential to be a natural modulator of APP processing and Aβ production in the brain.

Endoplasmic reticulum (ER) stress has been shown to be associated with neurodegenerative disorders including AD [7] and the ER chaperone protein, glucose-regulated protein 78 (Grp78) was demonstrated to bind APP and modify APP processing *in vitro* [8], [9], and was found in a complex with APP *in vivo* [10]. Since lactic acidosis is one of the many stressors that elicit an ER stress response, the effect of lactic acid on APP processing in the context of ER chaperone regulation was explored. We report here a novel role of lactic acid in activating an ER chaperone-APP interaction, modifying APP trafficking, increasing Aβ peptide generation and APP aggregate formation.

## Results

To study the effects of lactic acid on APP processing, we chose to use the human neuroblastoma cell line SH-SY5Y. These cells are known to express human amyloid precursor proteins and produce human Aβ peptides and have been widely used to study APP processing [11].

**Figure 1:**
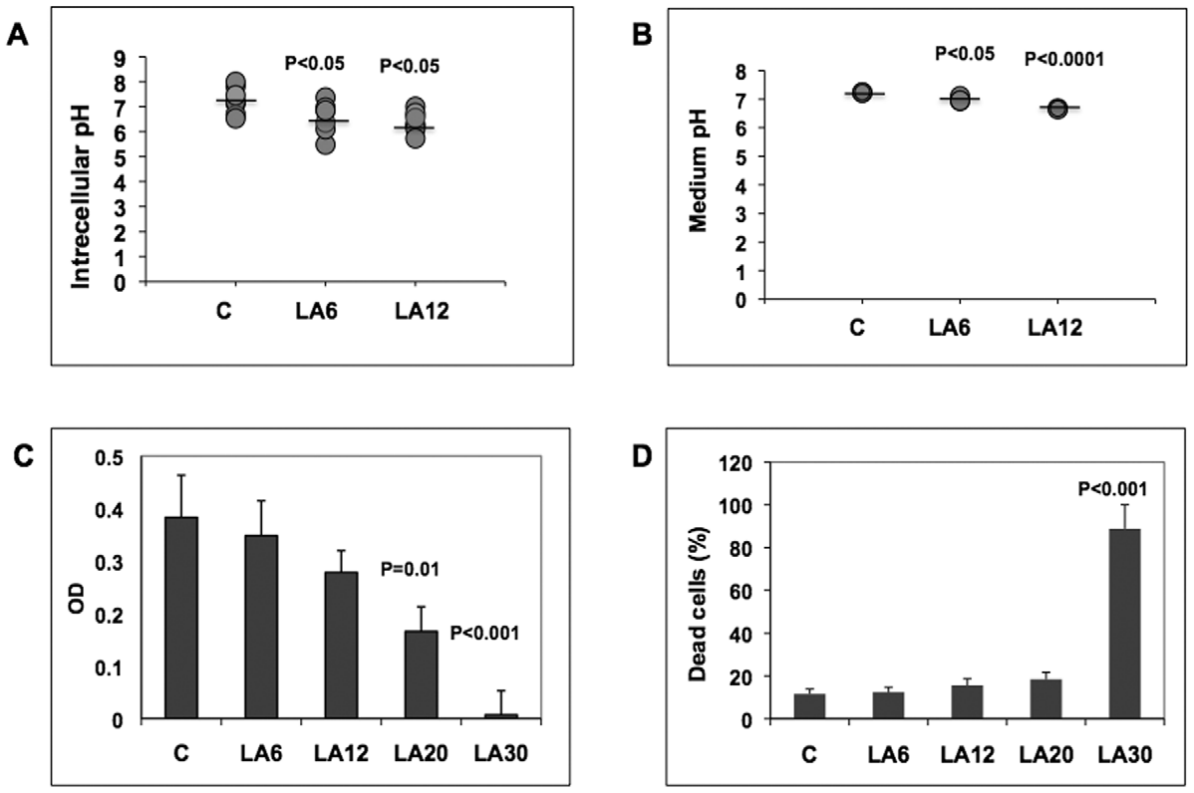
Lactic acid decreases extra- and intracellular pH. (A) Intracellular pH was measured using the pH-sensitive dye BCECF-AM. Fluorescence of SH-Y5Y cells exposed to 6 mM and 12 mM LA for 6 h and untreated control cells were scanned using a Leica TCS SP5 Laser Scanning Confocal Microscope. (B) The pH of culture media was measured with an UltraBasic UB-10 pH/mV meter (A. Daigger & Company, Chicago, IL, USA). Mean pH levels are noted by horizontal lines. (C) Viability of cells exposed to different concentrations of lactic acid was determined using an MTT Cell Viability Assay. (D) The percentage of dead (necrotic) cells at different concentrations of lactic acid was determined using Annexin V-FITC and Propidium iodide fluorescence.

First, the effect of lactic acid on the extra- and intracellular pH was determined. SH-SY5Y cells were incubated with a pH-sensitive dye (BCECF, AM) and changes in cell fluorescence upon exposure to lactic acid were determined using confocal microscopy. SH-SY5Y cells were exposed for 6 h to 6 mM and 12 mM lactic acid, which are concentrations within the physiological range of lactic acid measured in the blood at rest (∼1.5 – 4.5 mM) and shortly after strenuous exercise (∼10 – 14 mM) respectively [12], [13]. In the presence of 6 mM and 12 mM lactic acid, the cytosolic pH was lowered from pH 7.1 to pH 6.6 and 6.4, respectively (Fig. 1A), which paralleled a lowering of the pH in the cell culture medium (pH 7.2 under control conditions, pH 7.0 at 6 mM lactic acid and pH 6.7 at 12 mM lactic acid, respectively) (Fig. 1B). No substantial effects on cell viability were observed at these concentrations of lactic acid, although higher concentrations (up to 30 mM) did induce cell death (Fig. 1C, Fig. 1D).

**Figure 2:**
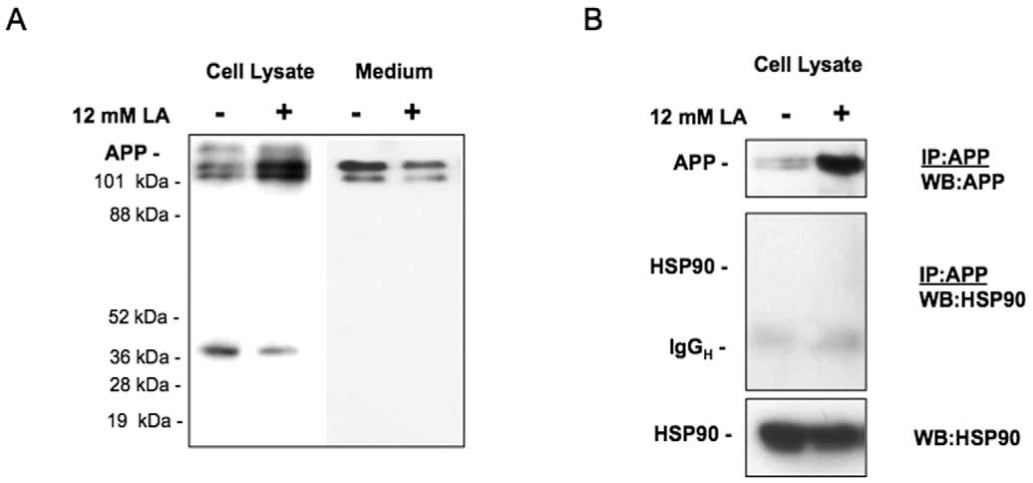
Lactic acid alters APP processing – lowering alpha secretase cleavage in SH-SY5Y cells. (A) APP processing was examined by immunoblot analysis of cell lysates and culture medium using the monoclonal antibody 6E10, which binds to amino acids 3-8 of APP (EFRHDS) and therefore detects full-length APP, sAPPα, and Aβ. Human SH-SY5Y neuroblastoma cells were cultured in the absence and presence of 12 mM lactic acid (LA) for 6 h. Accumulation of APP immunoreactivity in cells exposed to lactic acid and decreased levels of sAPPα in medium were detected. (B) Immunoprecipitation using 6E10 antibody followed by immunoblot confirmed the increased presence of APP in SH-Y5Y cells following exposure to 12 mM lactic acid (upper panel). An abundantly expressed control protein, heat shock protein 90 (HSP90), could not be detected in the immuno-precipitate (middle and lower panels).

**Figure 3:**
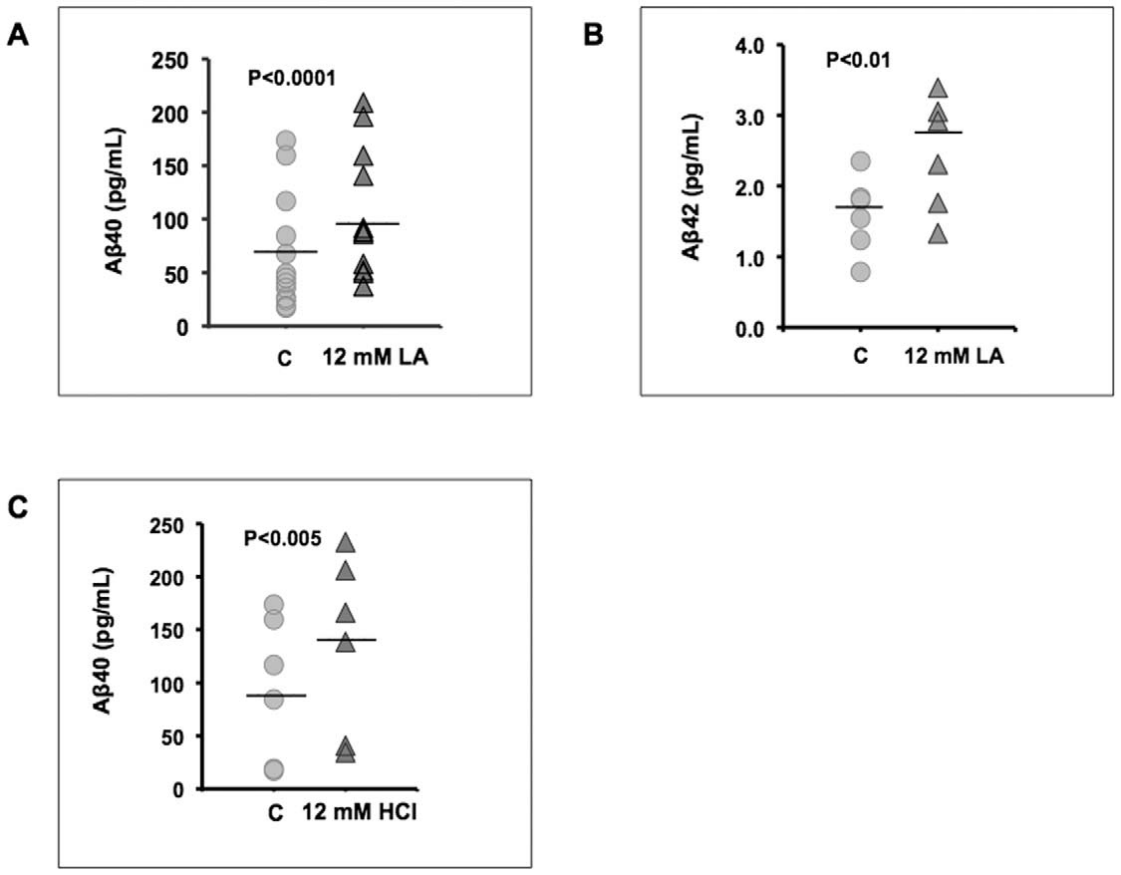
Lactic acid and HCl stimulate the secretion of Aβ40 and 42. The levels of Aβ40 (A) and 42 (B) were measured by ELISA in culture medium of SH-SY5Y cells exposed to 12 mM lactic acid for 6 h. (C) Aβ40 levels were measured in culture medium of SH-SY5Y cells exposed to 12 mM HCl for 6 h. The intracellular pH of cells exposed to 12 mM HCl dropped to approximately pH 5.5 (not shown). Mean levels of Aβ40 and 42 are noted by horizontal lines.

In an effort to gain insight into the cellular processing of APP in the presence of lactic acid, we determined the levels of APP in cell lysates and medium by immunoblot analysis using a monoclonal antibody, 6E10, which recognizes amino acids 3–8 of β amyloid (EFRHDS) and binds to full-length APP, the large extracellular domain of APP secreted upon APP cleavage by α-secretase (sAPPα?, and Aβ peptides [9]. SH-SY5Y cells cultured in 12 mM lactic acid for 6 h revealed a modest but consistent increase in the immunoreactivity of both, immature and mature forms of APP in cell lysates (Fig. 2A, left 2 lanes), and a decrease in the levels of secreted sAPPα(Fig. 2A, right 2 lanes). Immunoprecipitation confirmed an increase in APP upon treatment with lactic acid, but did not reveal association with the heat shock protein 90 (HSP90) (Fig. 2B), which had been implied in early amyloid β aggregation [14]. Small bands corresponding to Aβ peptides, however, were not detected in repeated western blot analyses and we therefore used ELISA assays as a more sensitive method for the detection of Aβ peptides. Indeed, 12 mM lactic acid increased the presence of Aβ4? peptides in cultured SH-SY5Y cells (Fig. 3A). A small but detectable increase in the secretion of Aβ42 was also noted (Fig. 3B). Upon lowering the extracellular pH to 5.5 by exposure to 12 mM HCl (data not shown), enhanced Aβ4? secretion was also observed (Fig. 3C). These results suggested that lactic acid affects Aβ peptide secretion. One possible mechanism could be the induction of BACE1 enzyme at lower intracellular pH levels, since HCl had a similar effect. Using commercially available kits to assay BACE1 activity (SensiZyme BACE1 Activity Assay Kit, Sigma, St. Louis, MO), we detected levels of BACE1 in SH-SY5Y cells that were just at the lower limit of detection and we did not observe that cells exposed to lactic acid possess levels of BACE1 activity that were elevated (data not shown). Notably, the assay system does not permit real-time measurements of BACE1 activity *in situ* and we are unaware of any assay that could. Thus, at the present time it is very difficult to know whether the lower intracellular pH in cells exposed to lactic acid is sufficient to increase BACE1 activity levels. The reported *ex vivo* data on BACE1 activity indicate that the greatest stimulation in activity occurs at pH 4 [6].

**Figure 4:**
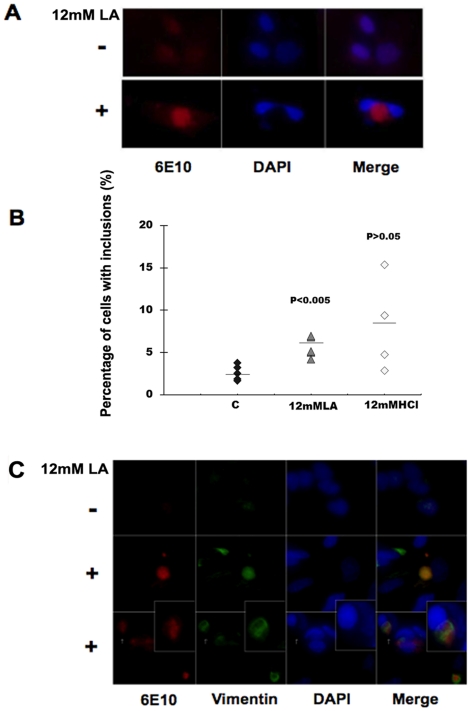
Lactic acid induces APP aggregation. (A) Fluorescence microscopy studies of SH-SY5Y cells were performed using the 6E10 antibody, which detects full-length APP, sAPPα, and Aβ peptides, respectively. Cells were untreated (Control) or exposed to 12 mM lactic acid (LA) for 24 h, fixed and stained with 6E10 and DAPI and examined by fluorescence microscopy. Perinuclear APP-positive material was detected in a subset of cells exposed to lactic acid. (B) Blinded evaluation of cells for the presence of APP-positive inclusions by two independent investigators revealed their presence in ∼2.5% of control cells, 6% of lactic-acid-treated and ∼8% of HCl-treated cells. (C) Fluorescence microscopy study of control and lactic acid-treated SH-SY5Y cells for APP (red) and intermediate filaments (green) using 6E10 and anti-vimentin antibodies, respectively. DNA was visualized with DAPI stain (blue). Following lactic acid treatment, the occurrence of vimentin filaments surrounding the inclusion-like structures reminiscent of aggresomes (inset) is observed.

Since the increase in intracellular APP was combined with a decrease of sAPPα in the culture medium, we hypothesized that lactic acid has an effect on APP processing through affecting its intracellular trafficking. Fluorescence microscopy studies were performed to determine the intracellular localization of APP in control and lactic acid-treated cells. Diffuse APP staining was observed in untreated SH-SY5Y (Fig. 4A, upper panels). In contrast, exposure of cells to 12 mM lactic acid for 24 h resulted in the perinuclear accumulation of APP immunoreactivity in a subset of cells (Fig. 4A, lower panels). Interestingly, perinuclear accumulation of APP was also observed in a subset of cells treated with 12 mM HCl, although this phenomenon was less consistent (Fig. 4B). The location of the APP containing structures was reminiscent of structures previously termed aggresomes [15]. A hallmark feature of aggresomes is the formation of an intermediate filament cage, primarily formed by vimentin, around the aggregated proteinaceous material. Indeed, co-immunostaining for vimentin and APP demonstrated the occurrence of vimentin filaments surrounding the inclusion-like structures (Fig. 4C, lower 2 rows and inset). Thus, a small percentage of SH-SY5Y cells accumulate APP in inclusion bodies with resemblance to aggresomes upon exposure to lactic acid. Although aggregates were also present in HCl-treated cells, we were unable to detect cage-like arrangement of vimentin in these cells.

**Figure 5:**
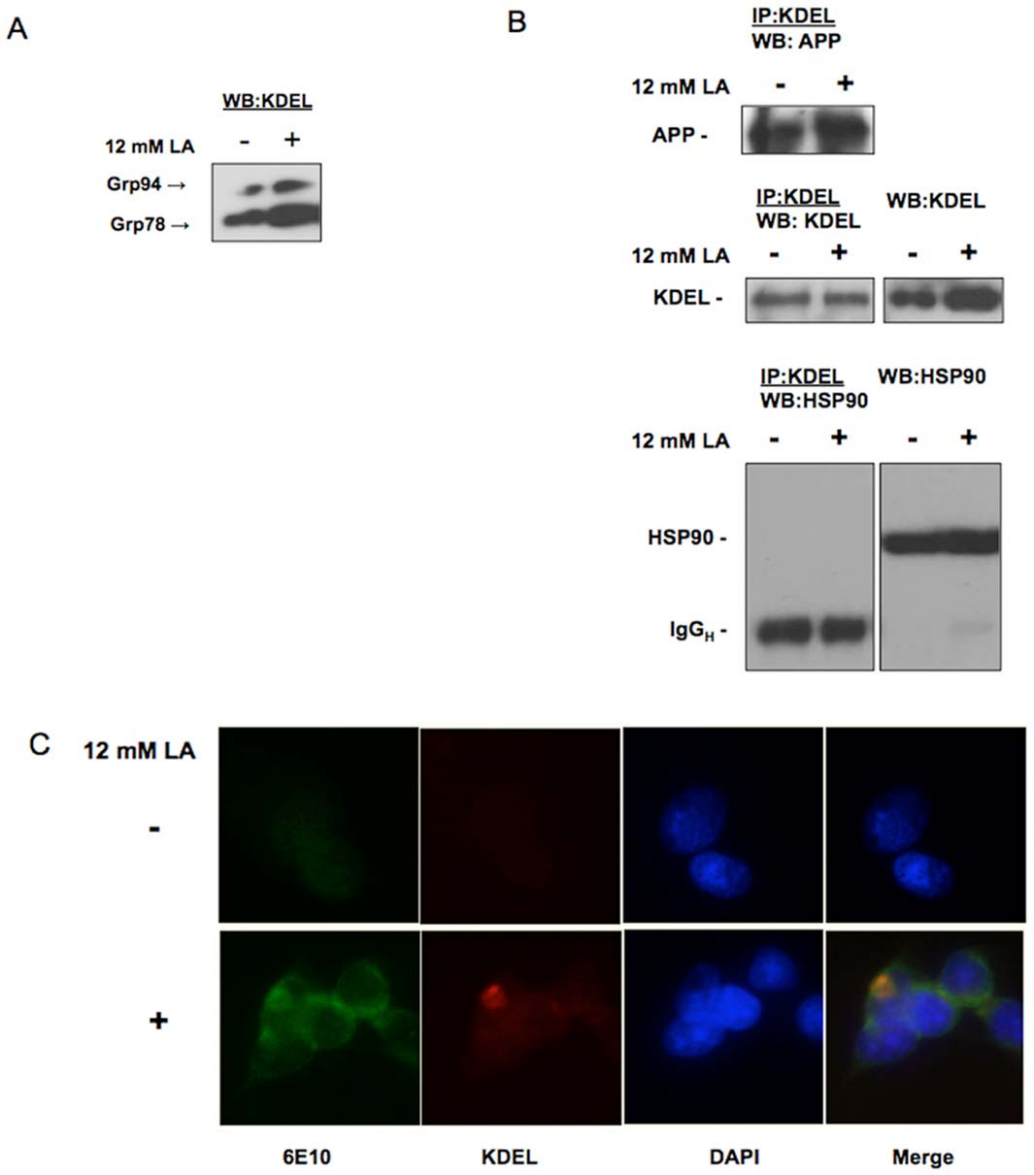
Lactic acid increases the expression of ER stress–dependent chaperone proteins Grp78 and Grp94 and enhances their interaction with APP. (A) Immunoblot analysis of SH-SY5Y cell lysates in the absence and presence of 12 mM lactic acid was performed using an antibody to the ER retrieval sequence KDEL, which is present in both, Grp78 and Grp94 proteins. Increased expression of Grp94 and to a lesser extent Grp78, was detected upon lactic acid treatment. (B) Immunoprecipitation using the anti-KDEL antibody revealed the increased presence of APP in complex with KDEL proteins following exposure of cells to lactic acid. An abundantly expressed control protein, heat shock protein 90 (HSP90, lower right panel), could not be detected in the immunoprecipitate (lower left panel). (C) Fluorescence microscopy study of APP using 6E10 (green) and KDEL proteins (red) documented co-localization (yellow) of APP and KDEL proteins in SH-SY5Y cells exposed to lactic acid.

Transient exposure to acidosis in the range of pH 6.4 to 6.0 has been shown to induce an ER stress response and increased expression of glucose-regulated protein 78, Grp78, one of the major ER-resident chaperone proteins [16], [17]. Grp78 was found to bind to APP both *in vitro* and *in vivo* [8], [9], [10]. To determine whether altered APP trafficking in lactic acid-treated cells could be related to the induction of an ER response, levels of Grp78 and Grp94 were determined. A substantial increase in the expression of Grp94, and to a lesser extent Grp78, was observed in lactic acid-treated compared to control SH-SY5Y cells (Fig. 5A). Furthermore, co-immunoprecipitation studies using an antibody to the KDEL epitope present in both, Grp78 and Grp94, revealed increased binding of KDEL proteins to APP of a single molecular weight corresponding to the immature form in lactic acid-treated cells (Fig. 5B, upper panel), but no binding to an abundantly expressed control protein, HSP90 (Fig. 5B, lower panel). Immunofluorescence microscopy documented a co-localization of APP and KDEL proteins in cells exposed to lactic acid (Fig. 5C). In HCl-treated cells, in contrast, APP-KDEL interactions were not observed.

Taken together these results suggest that lactic acid affects APP processing by the induction of an ER-stress response with increased expression of ER chaperone proteins Grp78 and Grp94 and that increased binding of Grp proteins to APP upon lactic acid exposure inhibits APP translocation from the ER to the Golgi compartment and alters APP processing by preventing exposure to α-secretase (on the cell surface and in the endocytic compartment), increasing exposure to BACE1 (in the ER/ER-Golgi intermediate compartment) and inducing APP aggregate formation by a mechanism not know at this point. It will be the subject of our further studies to determine, whether Grp protein interaction with APP is part of a cellular mechanism to prevent excessive APP aggregate formation in lactic acid-exposed cells or whether Grp protein binding to APP is part of a general unfolded protein response aimed at targeting improperly folded and/or aggregated proteins towards an ER-related degradation pathway.

## Discussion

In the present study, we demonstrate that human neuroblastoma cells, when exposed to physiologically relevant levels of lactic acid [12], which are within the range of lactic acid levels measured in the CSF of AD patients [5], secrete higher levels of Aβ40 and 42. Lactic acid lowered both the extra- and intracellular pH of these cultures, however, the overall pH remained well above the optimum for BACE1, which is reported to be at pH of 4 [6], and an effect on BACE1 activity could not be detected, suggesting alternative mechanism(s) underlying the altered APP processing in lactic acid-treated cells.

As lactate can be taken up by neurons as an energy source (for review see [18]), one hypothesis would be that the increased secretion of Aβ was due to a metabolic stimulation of neuronal activity, leading to increased protein synthesis and secretion [19]. Vantelon et al. reported that exposure of astrocytes to 16 mM lactate did not alter protein synthesis and at pH values below 6, protein synthesis was decreased [20]. In addition, in the presence of lactic acid a strong and sustained phosphorylation of the eukaryotic initiation factor 2α (eIF2α), the central regulator of the ER stress response, was reported [20]. We observed an increase in the expression of ER chaperone proteins Grp78 and Grp94 in SH-SY5Y neuroblastoma cells upon exposure to 12 mM lactic acid indicating the induction of an ER stress response; a hallmark of which is a reduction in general protein synthesis. Grp78 has previously been reported to interact with APP under pharmacologically induced ER stress conditions [9]. We now demonstrate that physiological lactic acid levels can elicit an ER stress response and the interaction of APP with Grp78/94 proteins and that this interaction leads to an alteration in APP processing documented by increased Aβ production, reduced sAPPα secretion, and retention and aggregation of APP.

Exercise is a very well characterized mechanism to increase blood lactate levels [12], and exercise has been demonstrated to play a protective role in AD [21]. Interestingly, studies in exercising healthy young subjects indicated that lactate taken up by the brain is metabolized since no increases of lactate in CSF or within the brain were detected [22]. In contrast, a significant release of lactate from the brain occurs under hypoxic conditions [23], [24], [25] and with extremely low blood pressure [26]; and lactic acid has clearly been shown to be elevated in the CSF of AD patients [5]. It is therefore conceivable, that during exercise, increased lactic acid in AD patients is metabolized and, based on our data, this would have a beneficial effect on disease progression.

A previous study by Brewer [27] also examined the effect of lactic acid on the processing of APP and reported increased accumulation of APP epitopes in primary rat hippocampal neurons treated with similar concentrations of lactic acid, but did not observe changes in secreted levels of Aβ. However, Brewer only examined cells that had been exposed to lactic acid for 4 days and we also observed that long-term incubations of cells with lactic acid (>48 h) did not produce statistically significant differences in secreted Aβ levels, but we also noted that this observation could be due to the cellular respiration in both control and lactic acid-treated cells which raised the levels of lactic acid in medium significantly over a 4 day period (data not shown).

It was reported previously that Grp78-APP interaction leads to a decrease in Aβ peptide secretion [8], [9], [28], whereas our data document increased Aβ generation concomitant with increased Grp-APP interaction in lactic acid-treated cells. It is important to point out that these studies used cells transfected with the Swedish mutation of APP (APPswe; K670N/M671L) together with either wild-type Grp78 or ATPase-mutant Grp78 (T37G), which functions as a molecular trap by stabilizing the normally transient interaction of polypeptides with Grp78; or used pharmacological induction of Grp78 overexpression. It is well established that APPswe distinguishes itself form wild-type APP by its altered subcellular localization of β-cleavage in the medial Golgi compartment compared to processing of wild-type APP in the endocytic compartment [29], [30], raising concerns that the use of APPswe could have masked potential effects of Grp chaperone protein binding on the subcellular processing of APP in these studies. In contrast, our study used a physiologically relevant concentration of lactic acid to stimulate neuroblastoma cells that endogenously express wild-type APP, small amounts of Grp94 and moderate amounts of Grp78 under unstimulated conditions.

Taken together our results suggest that rather than affecting BACE1 activity through altering cellular pH, lactic acid modulates APP processing via induction of an ER stress response and an increased interaction of APP with ER chaperone proteins and that this interaction contributes to the retention of APP in an ER/ER-Golgi-intermediate compartment, which leads to (i) reduced exposure to α-secretase and reduced sAPPα, and (ii) exposure to BACE1 leading to elevated Aβ levels. We can not rule out at this point that the differences in Aβ secretion upon APP interaction with KDEL proteins in our study and the studies previously reported is not based on the different nature of the interacting KDEL protein. Interaction of Grp78 with APP has been extensively reported [8], [9], but it is possible that APP binding to Grp94, which is readily induced by lactic acid treatment in our study, is involved in the observed increase in Aβ secretion.

Our results might have relevance for the use of therapeutics to reduce Aβ production, since aberrant subcellular APP processing in an early secretory compartment has recently been demonstrated to affect the efficacy of β-secretase inhibitors [30], and it needs to be further investigated whether the efficacy of β-secretase inhibitors is reduced in patients that are at risk for increased lactic acid levels in the brain.

## Materials and Methods

### Cell culture and measurements of Aβ, lactate, and cytotoxicity

Human neuroblastoma cells (SH-SY5Y) were cultured at steady-state of semi-confluency in 6-well plates in DMEM (Invitrogen, Carlsbad, CA), supplemented with 10% fetal bovine serum, 1 mM L-glutamine, and 100 U/ml penicillin and 100 mg/ml streptomycin at 37°C with 5% CO_2_. Media were harvested from cells that were placed in OPTI-MEM serum-free medium (Invitrogen, Carlsbad, CA) supplemented with lactic acid at 6 mM or 12 mM and incubated at 37°C for 6 or 24 h. Lactate concentrations in medium were detected using the Lactate Assay Kit from Biomedical Research Service Center (University at Buffalo, Buffalo, NY).

Aβ40 and Aβ42 ELISA assay kits were purchased from Invitrogen BioSource (Carlsbad, CA), protein assay kits from ThermoScientific-Pierce (Waltham, MA), and 6E10 monoclonal antibodies to APP and Aβfrom Covance (Princeton, NJ).

Cytotoxicity assays were performed using a MTT Cell Proliferation Assay Kit from Biotium, Inc. (Hayward, CA) and standard microplate absorbance readers, and an Annexin V-FITC Apoptosis Detection Kit from Abcam (Cambridge, MA).

### Measurement of extra- and intracellular pH

The pH of culture media was measured under incubator conditions (CO_2_ 5%, 37°C) with an UltraBasic UB-10 pH/mV meter (A. Daigger & Company, Chicago, IL, USA). Intracellular pH measurements were performed by incubating cells with pH-sensitive dye (BCECF, AM) following protocols provided by the manufacturer (Invitrogen, Carlsbad,CA). BCECF, AM was added to the culture medium to a final concentration of 5 µM. Cells were incubated for 15 min and then washed. Fluorescence was observed with a Leica TCS SP5 Laser Scanning Confocal Microscope running Leica LAS-AF software. Detection of BCECF fluorescence used a dual-excitation ratio with λ1 = 488 nm and λ2 = 465 nm and fixed emission at 558 nm.

### Immunoprecipitation

Immunoprecipitations (IPs) were performed using the Protein-G Immunoprecipitation Kit from Sigma (St. Louis, MO). Protein-G agarose beads were washed three times with IP buffer, incubated with 6E10 antibodies (Covance, Princeton, NJ) or KDEL antibodies (MBL Inc., Woburn, MA) for 1 h at room temperature. Two hundred micrograms of protein were added to 400 µl of IP buffer and 20 µl of protein-G agarose beads/primary antibody mixture. This mixture was gently mixed overnight at 4°C on a rocker. The agarose beads were collected by pulse centrifugation (5 seconds in the microcentrifuge at 13,000 rpm), the supernatant was discarded and beads were washed 5 times with 800 µl IP buffer. Finally, immunoprecipitated proteins were subjected to SDS-PAGE protein separation followed by western blot with APP and KDEL antibodies.

### Immunoblot

Immunoblotting was performed on cell lysates and medium proteins (20 mg protein per lane) separated by SDS-PAGE before transfer to nitrocellulose membrane (Amersham Bioscience, Uppsala, Sweden). After blocking in PBS with 5% milk, membranes were incubated overnight at 4°C with the primary antibodies; then washed and incubated with secondary antibodies conjugated to horseradish peroxidase (HRP), followed by incubation with Immobilon Western Chemiluminescent HRP Substrate (Millipore, Billerica, MA). The HSP90 antibody was purchased from Santa Cruz Biotechnology Inc. (Santa Cruz, CA).

### Immunocytochemistry

SH-SY5Y cells were grown in 4-well chamber slides. Cells were treated with 12 mM lactic acid for 6 h and subsequently fixed with 4% paraformaldehyde in PBS, washed and incubated for 72 h at 4°C with 6E10 primary antibodies at a dilution of 1∶100 in PBS containing 5% normal goat serum and 0.1% Triton X-100. After washing 5 times with PBS, slides were incubated with KDEL primary antibodies at a dilution of 1∶100 in PBS containing 5% normal goat serum and 0.1% Triton X-100 for 24 h at 4°C. Slides were washed 5 times in PBS and incubated consecutively with secondary anti-mouse IgG1-FITC antibodies and secondary anti-IgG2a-PE antibodies for 1 h at room temperature. The cover slips were washed 5 times with PBS, dried and mounted on slides. The slides were analyzed using a Zeiss Axioskop 2 fluorescence microscope (Carl Zeiss Microimaging Inc. Thornwood, NY) using the appropriate filters.

### Statistical Analysis

All experiments were performed at least 3 times. Data for measurements of pH levels and Aβ levels are presented as scatter plots and mean values. Statistical analyses used standard Student's t-test and differences between untreated and treated groups were deemed significant at p<0.05.

## References

[1] Steiner H (2008). The catalytic core of gamma-secretase: presenilin revisited.. Curr Alzheimer Res.

[2] Wolfe MS, Guenette SY (2007). APP at a glance.. J Cell Sci.

[3] Debray FG, Lambert M, Mitchell GA (2008). Disorders of mitochondrial function.. Curr Opin Pediatr.

[4] Schurr A (2002). Lactate, glucose and energy metabolism in the ischemic brain (Review).. Int J Mol Med.

[5] Redjems-Bennani N, Jeandel C, Lefebvre E, Blain H, Vidailhet M (1998). Abnormal substrate levels that depend upon mitochondrial function in cerebrospinal fluid from Alzheimer patients.. Gerontology.

[6] Lin X, Koelsch G, Wu S, Downs D, Dashti A (2000). Human aspartic protease memapsin 2 cleaves the beta-secretase site of beta-amyloid precursor protein.. Proc Natl Acad Sci U S A.

[7] Lindholm D, Wootz H, Korhonen L (2006). ER stress and neurodegenerative diseases.. Cell Death Differ.

[8] Yang Y, Turner RS, Gaut JR (1998). The chaperone BiP/GRP78 binds to amyloid precursor protein and decreases Abeta40 and Abeta42 secretion.. J Biol Chem.

[9] Kudo T, Okumura M, Imaizumi K, Araki W, Morihara T (2006). Altered localization of amyloid precursor protein under endoplasmic reticulum stress.. Biochem Biophys Res Commun.

[10] Bai Y, Markham K, Chen F, Weerasekera R, Watts J (2008). The in vivo brain interactome of the amyloid precursor protein.. Mol Cell Proteomics.

[11] Peraus GC, Masters CL, Beyreuther K (1997). Late compartments of amyloid precursor protein transport in SY5Y cells are involved in beta-amyloid secretion.. J Neurosci.

[12] Coco M, Di Corrado D, Calogero RA, Perciavalle V, Maci T (2009). Attentional processes and blood lactate levels.. Brain Res.

[13] Madureira G, Hasson-Voloch A (1988). Lactate utilization and influx in resting and working rat red muscle.. Comp Biochem Physiol A Comp Physiol.

[14] Evans CG, Wisen S, Gestwicki JE (2006). Heat shock proteins 70 and 90 inhibit early stages of amyloid beta-(1-42) aggregation in vitro.. J Biol Chem.

[15] Olzmann JA, Li L, Chin LS (2008). Aggresome formation and neurodegenerative diseases: therapeutic implications.. Curr Med Chem.

[16] Ladilov Y, Schafer C, Held A, Schafer M, Noll T (2000). Mechanism of Ca(2+) overload in endothelial cells exposed to simulated ischemia.. Cardiovasc Res.

[17] Aoyama K, Burns DM, Suh SW, Garnier P, Matsumori Y (2005). Acidosis causes endoplasmic reticulum stress and caspase-12-mediated astrocyte death.. J Cereb Blood Flow Metab.

[18] Quistorff B, Secher NH, Van Lieshout JJ (2008). Lactate fuels the human brain during exercise.. FASEB J.

[19] Brody DL, Magnoni S, Schwetye KE, Spinner ML, Esparza TJ (2008). Amyloid-beta dynamics correlate with neurological status in the injured human brain.. Science.

[20] Vantelon N, Rioux-Bilan A, Ingrand S, Pain S, Page G (2007). Regulation of initiation factors controlling protein synthesis on cultured astrocytes in lactic acid-induced stress.. Eur J Neurosci.

[21] Um HS, Kang EB, Leem YH, Cho IH, Yang CH (2008). Exercise training acts as a therapeutic strategy for reduction of the pathogenic phenotypes for Alzheimer's disease in an NSE/APPsw-transgenic model.. Int J Mol Med.

[22] Dalsgaard MK, Quistorff B, Danielsen ER, Selmer C, Vogelsang T (2004). A reduced cerebral metabolic ratio in exercise reflects metabolism and not accumulation of lactate within the human brain.. J Physiol.

[23] Siesjo BK (1982). Lactic acidosis in the brain: occurrence, triggering mechanisms and pathophysiological importance.. Ciba Found Symp.

[24] Gardiner M, Smith ML, Kagstrom E, Shohami E, Siesjo BK (1982). Influence of blood glucose concentration on brain lactate accumulation during severe hypoxia and subsequent recovery of brain energy metabolism.. J Cereb Blood Flow Metab.

[25] Norberg K, Quistorff B, Siesjo BK (1975). Effects of hypoxia of 10-45 seconds duration on energy metabolism in the cerebral cortex of unanesthetized and anesthetized rats.. Acta Physiol Scand.

[26] Feddersen K, Aren C, Nilsson NJ, Radegran K (1986). Cerebral blood flow and metabolism during cardiopulmonary bypass with special reference to effects of hypotension induced by prostacyclin.. Ann Thorac Surg.

[27] Brewer GJ (1997). Effects of acidosis on the distribution of processing of the beta-amyloid precursor protein in cultured hippocampal neurons.. Mol Chem Neuropathol.

[28] Hoshino T, Nakaya T, Araki W, Suzuki K, Suzuki T (2007). Endoplasmic reticulum chaperones inhibit the production of amyloid-beta peptides.. Biochem J.

[29] Thinakaran G, Teplow DB, Siman R, Greenberg B, Sisodia SS (1996). Metabolism of the “Swedish” amyloid precursor protein variant in neuro2a (N2a) cells. Evidence that cleavage at the “beta-secretase” site occurs in the golgi apparatus.. J Biol Chem.

[30] Yamakawa H, Yagishita S, Futai E, Ishiura  S beta-Secretase inhibitor potencyis decreased by aberrant beta-cleavage location of the “Swedish mutant” amyloid precursor protein.. J Biol Chem.

